# Supplementary material for: A Direct Interaction between Cyclodextrins and TASK Channels Decreases the Leak Current in Cerebellar Granule Neurons
Source: Biology (Basel). 2022 Jul 23;11(8):1097. doi: 10.3390/biology11081097 (PMC9331813; doi:10.3390/biology11081097)
Supplement: Supplementary file 1 [file biology-11-01097-s001.zip › biology-1799855-supplementary.pdf]

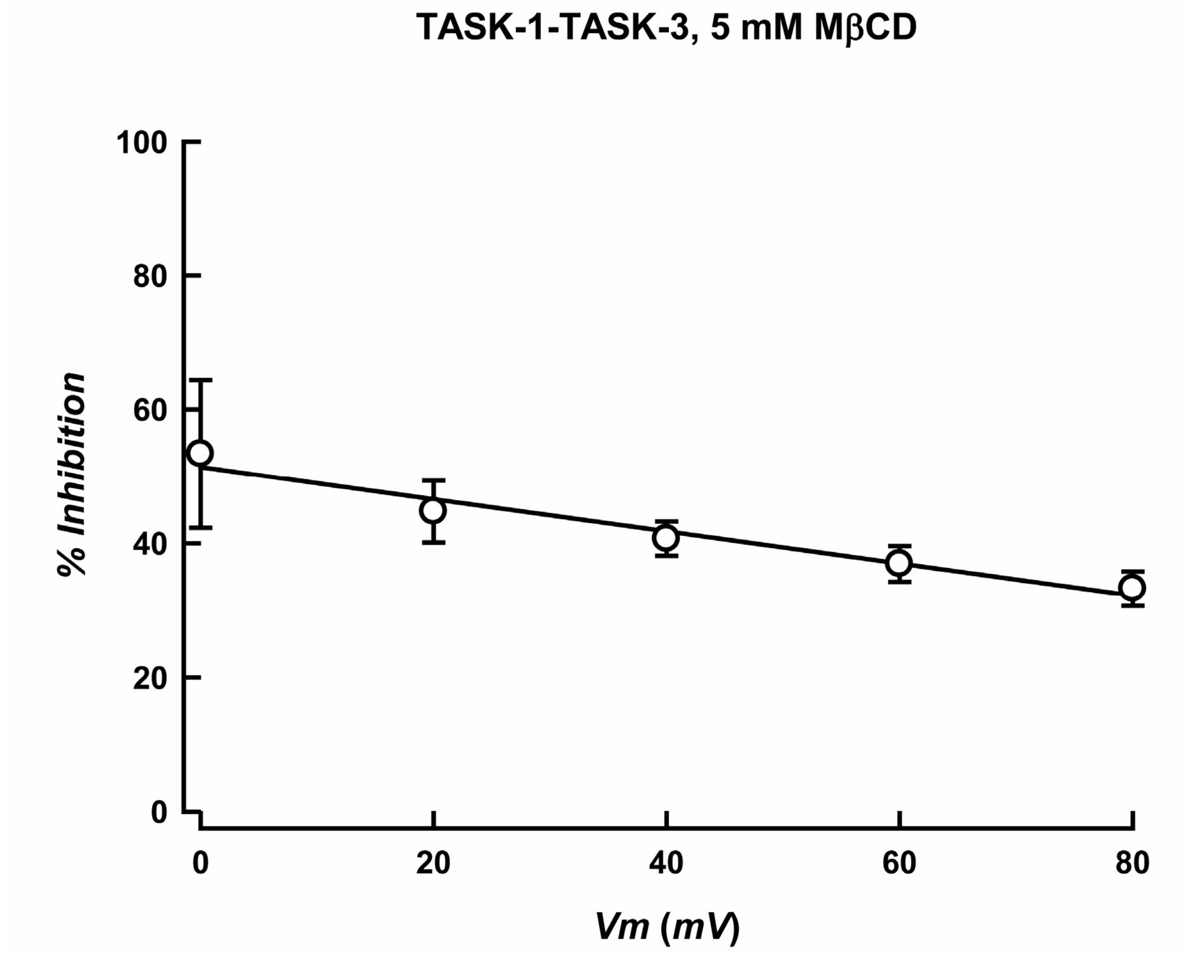

Figure S1: Methyl-β-cyclodextrin (MβCD)-mediated inhibition on concatenated construct TASK-1/TASK-3 currents expressed in HEK-293 cells.

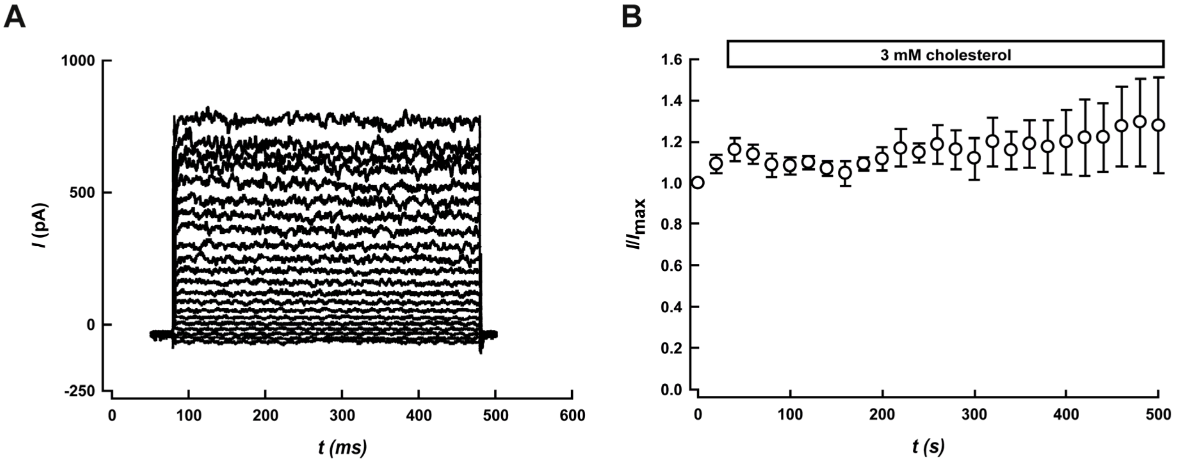

Figure S2: Effect of cholesterol perfusion on TASK-1/TASK-3 currents expressed in HEK-293 cells. (A) Representative current traces obtained for TASK-1/TASK-3 concatamer, expressed in HEK-293, using the voltage protocol described in Figure 5. (B) Time course of treatment with cholesterol on the current measured at +60 mV. Results are means ± SEM (n = 4 experiments).
